# Preferences for Very Low and Very High Voice Pitch in Humans

**DOI:** 10.1371/journal.pone.0032719

**Published:** 2012-03-05

**Authors:** Daniel E. Re, Jillian J. M. O'Connor, Patrick J. Bennett, David R. Feinberg

**Affiliations:** Department of Psychology, Neuroscience and Behaviour, McMaster University, Hamilton, Ontario, Canada; University of Sussex, United Kingdom

## Abstract

Manipulations of voice pitch have been shown to alter attractiveness ratings, but whether preferences extend to very low or very high voice pitch is unknown. Here, we manipulated voice pitch in averaged men's and women's voices by 2 Hz intervals to create a range of male and female voices speaking monopthong vowel sounds and spanning a range of frequencies from normal to very low and very high pitch. With these voices, we used the method of constant stimuli to measure preferences for voice. Nineteen university students (ages: 20–25) participated in three experiments. On average, men preferred high-pitched women's voices to low-pitched women's voices across all frequencies tested. On average, women preferred men's voices lowered in pitch, but did not prefer very low men's voices. The results of this study may reflect selection pressures for men's and women's voices, and shed light on a perceptual link between voice pitch and vocal attractiveness.

## Introduction

Preferences for stimuli that exaggerate sexually-selected features are found throughout the animal kingdom. For example, female sticklebacks prefer artificial mates that are larger than naturally occurring males [1], male silver-washed fritillary butterflies are attracted to abnormally rapid movement of females' wings [2], female canaries are drawn to songs with synthetically enhanced syllable rates [3], and female stalk-eyed flies prefer males with unnaturally long eye-stalks [4].

One trait in humans that appears to have undergone intense sexual selection is the average fundamental frequency of the voice [5]. The perception of fundamental frequency and corresponding harmonics is commonly known as voice pitch. While voice pitch reflects allometric scaling in several primate species [6], [7], humans appear to be unique among primates in that sexual dimorphism of adult voice pitch is well beyond what may be explained by height alone [8], [9], [10]. The human larynx is made of cartilage and muscle that are rich in androgen receptors and grow rapidly during puberty [11], [12]. Men's vocal cords lengthen and thicken much more so than women's, resulting in the adult male voice pitch being on average half the frequency of adult female voice pitch [11]. Historical records of male singers that were castrated before puberty indicate that voice pitch does not reach adult male levels in the absence of testicular hormones at puberty [13]. Similarly, case studies of male-to-female transsexuals indicate that although women may have different quantities and sensitivities of androgen receptors on their vocal cords, administering exogenous testosterone to women does lead to a drop in voice pitch [14]. Furthermore, after menopause, the pitch of many women's voices drops, most likely due to a decrease in estrogen production and an increase in testosterone production [15], [16]. Thus, voice pitch is indicative of laryngeal development, and is dependent on pubertal and fluctuating levels of sex hormones [11], [16].

Several empirical studies have found that in general, women prefer lower-pitched men's voices to higher-pitched men's voices [17], [18], [19], [20], [21], [22], [23]. Men with lower-pitched voices also have higher reproductive success than men with higher-pitched voices [24]. Conversely, men typically prefer high-pitched women's voices over low-pitched women's voices [25], [26], [27]. High-pitched women's voices are perceived as feminine and youthful [25], [27] and flirtatious [28], and increase the perceived likelihood of sexual infidelity [29]. Furthermore, women tend to raise their voice pitch when presented with an attractive man's face and asked to leave a phone message for him [30].

In general, men prefer high-pitched women's voices whereas women prefer low-pitched men's voices, however two studies obtained opposing results when analyzing men's preferences at the upper limit of the natural frequency range of women's voices [27], [31]. Specifically, one study found that men prefer these very high-pitched voices [27] whereas the other study shows that men did not prefer these voices [31]. Therefore it is important to further investigate whether men and women prefer very high and very low voice pitch, respectively. Determining if people exhibit preferences for voice pitch beyond that fall below or above (very low or very high) what is normally produced in a population may shed light on whether preferences for men's and women's voice pitch are directional or stabilizing. Preferences for very low pitch in men's voices and/or very high pitch in women's voices may suggest directional preferences [32], but a lack of such preferences for such extreme voice pitches may indicate stabilizing preferences.

The ability to discriminate vocal frequencies also may constrain men's preferences for high-pitched women's voices. The relationship between physical and perceived frequencies is logarithmic such that as physical frequency increases, the ability to hear the difference between frequencies diminish, thus making it more difficult to discriminate high frequency voices than low frequency voices [33]. Men's preferences for women's voices may therefore be limited by two non-mutually exclusive adaptive mechanisms to negate disparities in physical and perceived frequencies and avoid poor mate-choice decisions: (1) a cut-off point where men are averse to very high-pitched women's voices, ensuring formant frequency discrimination is not limited by voice pitch; (2) ensuring the difference in pitch required to change attractiveness ratings is tied to logarithmic pitch perception. Here we would expect larger just-noticeable differences (JNDs) in attractiveness ratings than for pitch discrimination to minimize error in potentially costly mate-choice decisions.

The current experiments tested for perceptual boundaries on preferences for voice pitch, and examined whether preferences for voice pitch are directional or stabilizing. We manipulated men and women's voices in 2 Hz intervals of voice pitch from above and below the normal range of men and women's voices and presented each voice to opposite-sex participants to measure voice preferences.

## Methods

Protocols for this study were approved by the McMaster University Research Ethics Board. All participants gave written informed consent prior to participating.

### Stimuli

**Figure 1:**
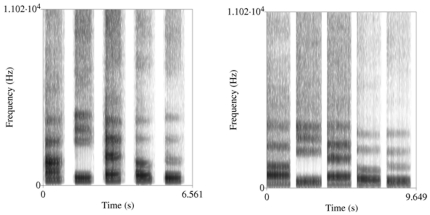
Spectrograms of the average female (left panel) and male voice (right panel) for the five vowel sound stimuli. Spectrograms plot time on the X axis, frequency on the Y axis, and amplitude is represented by shading.

To create voices representative of a given population, we created an initial male and female voice speaking the English monopthong vowel sounds: ‘eh’ as in ‘bet’, ‘ee’ as in ‘see’, ‘ah’ as in ‘father’, ‘oh’ as in ‘note’, and ‘oo’ as in ‘boot’. Such stimuli have been used in many studies on voice perception [17], [22], [27], [34], [35]. Initial voices were created from an average of 32 male (mean: 109.99 Hz, SD: 3.18 Hz, range: 86–152 Hz) and 32 female voices (mean: 210.81 Hz, SD: 20.67 Hz, range: 143–285 Hz), separately, using STRAIGHT [36]. Briefly, this procedure entails pitch extraction, and demarcating key spectral features (e.g., formant frequencies and vowel onset and offset) on spectrograms of the sound (Figure 1). These features are then aligned in time, and then fundamental frequency and harmonics, amplitude, time, and formant frequencies are then averaged separately, and voices reconstructed. This method has been used successfully in other studies of voice processing [37]. The averaging process averaged voices in pairs, iteratively, until one base voice of each sex were created from an average of 32 voices. The final pitches of the averaged voices were 110 Hz for the male voice and 211 Hz for the female voice. Spectrograms of the average male and female voice used in this study are shown in Figure 1.

Next, we manipulated voice pitch using the Pitch-Synchronous Overlap Add (PSOLA) algorithm [38] in Praat acoustic phonetics software [39]. The initial voices were manipulated in 2 Hz steps using the PSOLA method. The PSOLA method selectively manipulates mean fundamental frequency and corresponding harmonics independent of time and formant frequencies, and has been used successfully in many studies on voice preferences and other mate-choice relevant contexts in humans [17], [21], [22], [27], [40], [41], and other mammalian species [42], [43]. Although voice pitch was manipulated, formant frequencies were retained, and previous research has demonstrated that such manipulations create voices that still sound “adult-like” [35]. The pitch range for men's voices was 60–180 Hz, and the pitch range of women's voices was 160–300 Hz. These pitch ranges extend well below the 32 men's voices and above the 32 women's voices used in creating the initial averaged voices. Praat's pitch parameters were set at a minimum 50 Hz and maximum 300 Hz for men's voices, and a minimum 100 Hz and maximum 600 Hz for women's voices. Window length was determined automatically by Praat.

### Participants and procedures

Ten men (mean age: 21.80, SD: 1.45) and nine women (mean age: 22.05, SD: 1.58) participated in the study. All were university students with normal hearing. All participants reported English as their first language, and no participant reported any musical training. Participants rated voices in a 2-alternative forced-choice paradigm: on each trial, two voices were presented, one after the other. Participants were free to replay the voices as desired. Voice trials were presented using the method of constant stimuli [44]. In this method, each voice pitch was compared to every other pitch in random order. The method of constant stimuli was chosen to avoid auditory adaptation to stimuli which may have affected JNDs. Furthermore, extensive sampling of the pitch dimension allowed for an in-depth analysis of possible perceptual constraints on preferences for voice pitch. Extensive sampling of few participants is common practice in auditory psychophysics [45], [46], [47], [48], [49], [50], [51].

Men listened to all possible pairs of women's voices, and women listened to all possible pairs of men's voices. Catch trials (i.e., no difference in pitch) at each pitch interval were included. Men listened to 51 blocks of 50 voice pairs and 1 block of 6 voice pairs, while women listened to 37 blocks of 50 voice pairs and 1 block of 42 voice pairs. Each block of 50 voice pairs took approximately 15 minutes to complete, and participants completed a maximum of eight blocks per day. Each task took several weeks to complete. The frequencies of all voices were randomized within and between blocks. In all, males listened to 2556 voice pairs, and females listened to 1892 voice pairs.

The study was divided into three tasks. The first task was a simple pitch discrimination task. Four men and four women were asked to pick the voice with the higher pitch [47]. Several previous studies have assessed JNDs in pitch for vowel sounds [45], [46], [52], [53].

The second task was created to determine JNDs in vocal attractiveness based on pitch manipulations. Four men and four women listened to all the voice trials and were asked to pick the voice they thought was more attractive. Two men and three women who completed the pitch discrimination task also completed the voice attractiveness task. As tasks were performed months apart, it is unlikely the voice attractiveness task affected performance on the pitch discrimination task.

The third task assessed JNDs in voice pitch for perception of vocal dimorphism (masculinity or femininity). Four men and four women participated in the third experiment. Men were presented with pairs of women's voices and were asked to choose the voice they thought was more feminine (as in [27]). Women were presented with pairs of men's voices and were asked to choose which voice they thought was more masculine.

## Results

### Pitch discrimination task

Psychometric functions were created for each participant by plotting the proportion of correct responses as a function of the Weber fraction (the difference in pitch between the two voices in a trial divided by the lower pitch value). Regression was used to estimate the best-fitting logit or probit function to each participant's data. Catch trials were not included in the psychometric functions, but were used as an index of side bias that was included as a predictor in the regression model. The JND was defined as the Weber fraction that produced correct responses on 75% of trials [54]. Individual JNDs for each participant can be seen in Table 1. The regression model provided good fit to the data, and accounted for a significant amount of variance for each participant (χ^2^>158.0, p<0.01 in all cases). We failed to find a significant difference between men's and women's pitch discrimination thresholds (independent-sample t-test: t(6) = 0.54, p = 0.61, Cohen's d = 0.38). Thus, we collapsed our analysis across sexes. The average JND for pitch discrimination across the eight participants was 4.1% (SD: 1.9%). See Table 1 for all averaged JNDs.

**Table 1:** Individual and average just-noticeable differences for men and women in all three tasks.

Judgment	Sex of participant and voice	Average JND (%)
Pitch discrimination	Men rating women and women rating men	**4.1**
Vocal attractiveness	Men rating women	**18.2**
Vocal attractiveness	Women rating men	**8.8**
Vocal femininity	Men rating women	**5.6**
Vocal masculinity	Women rating men	**6.5**

### Vocal attractiveness task

**Figure 2:**
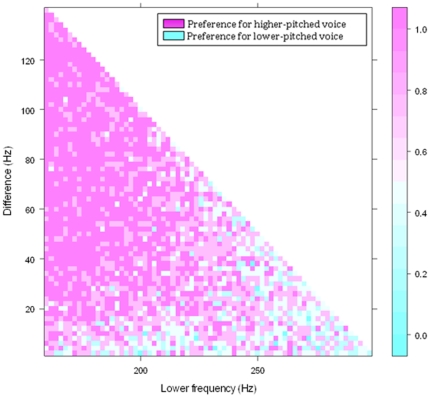
Men's vocal preferences as a function of lower frequency (Hz) and the difference between two voices (Hz) in a voice trial. Men preferred higher-pitched voices across the range of women's frequencies where the stimuli difference is above the JND in attractiveness of 18.2%. Preferences were averaged across all four male participants.

Psychometric functions were created for each participant in the vocal attractiveness task by plotting the proportion of trials a subject preferred the higher-pitched voice as a function of the Weber fraction, and fitting logistic or probit functions to the data. Just-noticeable differences were determined for each participant. Men's and women's JNDs were analyzed separately. For men rating women's voices, the JND was defined as the Weber fraction at which participants rated the higher-pitched voice as more attractive on 75% of trials [54]. Regression analyses revealed a significant association between attractiveness and the Weber fraction for each male participant (χ^2^>155.8, p<0.01 in all cases). The JNDs of the four male participants were averaged to determine an average JND of 18.2% (SD: 8.6%). Preferences for high-pitched women's voices increased monotonically with Weber fraction, and were maintained across the entire range of presented frequencies (Figure 2).

To test whether there were limits to how high or low voice pitch could be manipulated and still sound attractive, we created linear and quadratic models of preference strength as a function of Weber fraction and lower frequency. We then assessed the goodness of fits of the linear and quadratic models. If there are directional preferences for high-pitched women's voices, preference strength would be best fit by a linear model. If there are stabilizing preferences, preference strength would be best represented by a “U” shaped quadratic model in which the apex of the “U” occurs where preference strength is weakest. Both the linear (F(1, 2484) = 785.7, p<0.01) and the quadratic (F(2, 2484) = 393.0, p<0.01) models were significant. We determined which model provided a better fit to the data by using Akaike's Information Criterion [55], which is sensitive to residual error as well as the number of parameters used in each model. The information criterion test revealed an Akaike's Information Criteria of 2 (quadratic-linear), giving a 73% probability that the linear model was the better fit. The linear model was therefore 2.7 times more likely to produce a better fit than the quadratic model. Thus, stabilizing preferences are less likely than directional preferences given the range of frequencies tested here.

**Figure 3:**
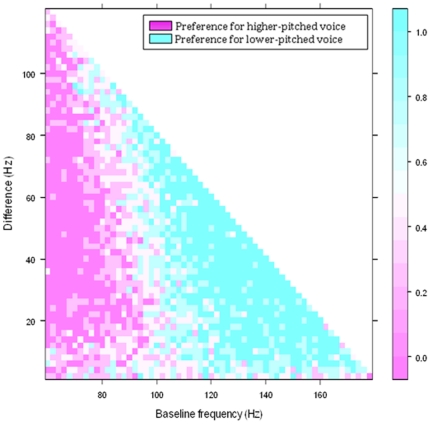
Women's vocal preferences as a function of lower frequency (Hz) and the difference in between two voices (Hz) in a voice trial. Women preferred lower-pitched voices in trials where the lower-pitched voice was above ∼96 Hz, however, they preferred the higher-pitched voice in trials where the lower-pitched voice fell below ∼96 Hz. Preferences were averaged across all four female participants.

For women rating men's voices, the JND was defined as the Weber fraction at which participants rated the lower-pitched voice as more attractive 75% of the time [54]. Regression analyses revealed a significant association between attractiveness and the Weber fraction for each participant (χ^2^>30.7, p<0.01 in all cases). However, women's preferences for the lower-pitched men's voices were not a monotonic function of frequency. Instead, women preferred low-pitched voices on trials that contained a lower frequency voice that was greater than 96 Hz, but preferred the higher-pitched voice on trials in which the lower voice frequency was less than 96 Hz (Figure 3). Thus, women did not prefer low-pitched men's voices on trials in which the lower frequency was extremely low.

Again, both linear and quadratic models of preference strength were produced to empirically test whether preferences were directional or stabilizing. Stabilizing preferences would be best represented by a “U” shaped quadratic model where the apex of the “U” shape occurs at the inversion in preferences. Directional preferences would best be represented by a linear model. The quadratic model was significant (F(2, 1823) = 19.2, p<0.01), however the linear model was not significant (F(1, 1823) = 1.44, p = 0.23). We tested which model better fit the data via Akaike's Information Criterion. The information criterion test revealed an Akaike's Information Criteria of −35.6 (quadratic-linear), giving a 99.99% probability that the quadratic model was the better fit. The quadratic model was therefore 54 027 298 times more likely to produce a better fit than the linear model. Thus, the data strongly suggest that women's preferences for voice pitch are stabilizing, such that women prefer voices raised in pitch to very low pitch.

Due to the fact that preferences were not a monotonic function of frequency, JNDs for women's ratings of men's vocal attractiveness were calculated using only responses collected with voice frequencies ranging from 110 Hz to 180 Hz. Setting the lowest pitch to 110 Hz, rather than 96 Hz, ensured that any residual preferences for the higher-pitched voice was excluded. The shortened pitch range still contained 666 trials, which is sufficient to create an accurate psychometric function. Within this range of frequencies, preference for the lower-pitched voice was related monotonically and significantly to the Weber fraction for each participant (χ^2^>30.65, p<0.01 in all cases), and the average JND for women rating men's voices for attractiveness was 8.8% (SD: 2.7%). The difference between JNDs for men's and women's ratings of attractiveness approached significance (independent-sample t-test: t(6) = 2.05, p = 0.08, Cohen's d = 1.45).

### Vocal dimorphism task

For men rating women's voices, the JND was defined as the Weber fraction at which participants rated the higher-pitched voice as more feminine on 75% of trials [54]. The regression analyses revealed a significant association between perceived dimorphism and the Weber fraction for all participants (χ^2^>125.7, p<0.01 in all cases). The average JND for men rating women's voices for femininity was 5.6% (SD: 4.9%).

For women rating men's voices, the JND was defined as the Weber fraction at which participants rated the lower-pitched voice as more masculine on 75% of trials [54]. The regression analyses revealed a significant association between perceived dimorphism and the Weber fraction for all participants (χ^2^>152.0, p<0.01 in all cases). The average JND for women rating men's voices for masculinity was 6.5% (SD: 4.3%).

### Comparison of JNDs across tasks

The JNDs for pitch discrimination, vocal attractiveness, and vocal dimorphism were compared. The JNDs for pitch discrimination were collapsed across sex (8 JNDs), as there were no significant differences in men's and women's pitch discrimination. The JNDs for vocal attractiveness and vocal dimorphism were kept within-sex (4 JNDs).

A Welch independent sample t-test was used to assess differences between pitch discrimination and ratings of vocal attractiveness. A Welch t-test was used as the groups did not satisfy the assumption of equal variance. The t-test revealed significant differences between JNDs for pitch discrimination and men's ratings of attractiveness (t(3.2) = 3.20, p<0.05, Cohen's d = 2.24). An independent samples t-test revealed a significant difference between pitch discrimination and women's ratings of vocal attractiveness (t(10) = 3.45, p<0.01, Cohen's d = 1.93).

Independent-sample t-tests failed to find significant differences between the pitch discrimination and men's ratings of vocal femininity (t(10) = 0.73, p = 0.48, Cohen's d = 0.38) or between pitch discrimination and women's ratings of vocal masculinity (t(10) = 1.36, p = 0.20, Cohen's d = 0.72).

An independent-sample t-test revealed a significant difference between men's ratings of vocal attractiveness and men's ratings of vocal femininity (t(6) = 2.54, p = 0.04, Cohen's d = 2.12). An independent-sample t-test did not reveal a significant difference between women's ratings of vocal attractiveness and women's ratings of vocal masculinity (t(6) = −0.90, p = 0.40, Cohen's d = 0.64).

## Discussion

We found that men preferred high-pitched women's voices to low-pitched women's voices, even when voice pitch was above the normal speaking range, and our analyses suggest that men's preferences for high voice pitch in women may contribute to directional selection on women's voice pitch. These data also support previous findings that men prefer high-pitched women's voices to low-pitched women's voices [26], [53], [56], [57], [58], [59], [60]. Such preferences for very high voice pitch among women may be adaptive as voice pitch is indicative of potential reproductive health [61] and youth. Some women's voices may naturally fall below the 160 Hz minimum we used in the current study, however the higher-pitched voice was preferred in all trials presented, and it is unlikely preferences would invert below the pitch range studied. We used women's voices with pitches as high as 300 Hz; higher than the voice pitch range among women in our sample (143–285 Hz) however it is possible that men do not prefer voice pitch higher than the frequencies tested here. Indeed, above 300 Hz, there exists the possibility that the fundamental frequency of manipulated voices could be higher than the first formant frequency, creating speech-like sounds that are physically impossible for humans to make. That did not occur in the stimuli used in our experiments.

We found that, on average, women preferred a lower pitched voice over a higher pitched voice except when the lower frequency was below ∼96 Hz, in which case women tended to prefer the higher pitched voice. While a natural pitch of 96 Hz or lower can be found in some men, it is rare and falls outside the standard deviation of the frequencies used to create stimuli in the current experiment. Thus, women prefer low pitch in men's voices, but not very low pitch. Such preferences may contribute to stabilizing selection pressure for low pitch in men's voices [32]. One possible mechanism underlying this constraint may be the anatomical properties of the vocal cords themselves. Extremely low voice pitch may be indicative of pathology, or laryngeal damage caused by smoking [62], [63]. Such voice pitch may also be indicative of overspending of resources on producing a large larynx, such as in hyperpituitarism [64]. Voices below 70 Hz may even sound unnaturally “pulsed” rather than modal [11]. Furthermore, the perception of vowel sounds is attenuated when fundamental frequency is too low [47], and rich semantic and emotional information given by intonation in speech and singing may be difficult to perceive at such low frequencies [11].

The female participants in the current study were all university students in their early twenties. Previous research has demonstrated that women's voice preferences are developed in adolescence [20], however it is possible that preferences change with age. Indeed, men's voice pitch does change throughout adult development [65], [66], and preferences may change as a form of age-related assortative mating (as has been found in other mammals, [67]). Age-related changes in men's voices are relatively minimal (∼6–8 Hz, [65], [66]), however, and the preference limit on low-pitched men's voices was around 96 Hz, well below average male voice pitch and beyond changes in pitch associated with age. Thus, it is unlikely that participant age affected the limitation on preferences for low-pitched men's voices found here.

We found in both sexes that preferences for voice pitch varied logarithmically with respect to physical pitch, indicating a perceptual link between preferences for voice pitch and physical frequency of the voice itself. Feinberg et al. [27] found that raising and lowering the pitch of female voices by 20 Hz had a stronger effect on voices that were low in pitch before being manipulated than on voices that were average-pitched or high-pitched before manipulation. These effects, however, were not found for femininity ratings. Thus, it was unknown whether the diminishing effect of changes in voice pitch on attractiveness as the lower voice pitch increased was due to the Weber-Fechner Law, or a maximum frequency that men find attractive in women's voices. The research reported here suggests preferences for voice pitch are influenced by the Weber-Fechner Law.

Just noticeable differences in preferences for voice-pitch were significantly higher than both JNDs for masculinity/femininity ratings and pitch discrimination. This may be due to the difference in the degree of neural processing involved in the pitch discrimination and vocal attractiveness tasks. Pitch discrimination requires a straightforward decision on pitch intensity [68], [69] and involves voice-selective neural systems [70]. Judgments of attractiveness likely require involvement from neural reward centers [71] and neural areas involved in processing speaker identity [72]. The JNDs in vocal dimorphism, however, were not significantly different from those for pitch discrimination. Thus, the differences in JNDs between tasks cannot be attributed to pitch discrimination having an objectively correct answer while social attributions do not. The perceptual link between pitch and attractiveness may be less tightly coupled because vocal attractiveness is based on several vocal parameters besides pitch, including formant frequencies [17], [23], [53], [73], intonation [74], and word content [23], [40]. Regardless of the proximate mechanism, poor mate-choice decisions can be costly for both men and women [75], and thus it may be functionally adaptive for JNDs in attractiveness to be relatively larger than JNDs for pitch discrimination.

Several studies have assessed JNDs in pitch for spoken sounds and have found discrimination thresholds ranging from 1–2% [45], [46], [51] for synthetic vowel sounds to 6% for spoken vowel sounds [53] and 7% for naturalistic sentences [73]. Pisanksi and Rendall [53] described how JNDs may be larger for vocalized vowel sounds than synthetic vowel sounds due to increased spectral variation in human vocalizations. Another reason why our JNDs in pitch discrimination are higher than previous studies is that participants in our study made assessments after hearing an entire series of vowels, rather than making assessments after individual vowels as in other studies [45], [46], [51]. Indeed, JNDs for pitch discrimination are much higher for sentences than individual vowels [73], suggesting that the added pitch variation over a longer utterance can make it harder to discriminate average pitch between utterances.

The current study used digitally averaged voices. Previous research has demonstrated that averaged voices can be perceived as more attractive than component voices, due to reducing vocal aperiodicties that can be present in any individual voice [76]. Thus, the initial averaged voices may be more attractive than their component voices. All stimuli in the current study were products of averaged voices, which were then compared against each other to determine preference limitations and JNDs. Since the current experiments pitted averaged voices against other averaged voices, we see no reason why relative pitch preferences and JNDs should not be generalizable, though it is possible results using natural voices may vary. It is also important to note that formant frequencies for the averaged voices were retained after digital pitch manipulations. Formant frequencies interact with pitch to affect judgments of size [47] and attractiveness [77], thus manipulating formants along with pitch may affect the current results.

In summary, our results demonstrate that men prefer high-pitched women's voices at very high frequencies, while women do not prefer low-pitched men's voices at very low frequencies. This may reflect different selection pressures acting on voice pitch in men and women. Just-noticeable differences in perceived vocal attractiveness are significantly greater than those needed to discriminate pitch, which in turn were not significantly different from those needed to alter perceived vocal masculinity and femininity. If our ancestors also exhibited these perceptual constraints on the relationship between voice pitch and vocal attractiveness, this may have helped shape the evolution of sex differences in voice pitch.
